# Comparison between the General Assessment of Hospitalised Patient Tool and the Barthel Index: A Retrospective Study

**DOI:** 10.3390/nursrep13030100

**Published:** 2023-08-23

**Authors:** Matteo Danielis, Sara Bortot, Renzo Zanotti

**Affiliations:** Laboratory of Studies and Evidence Based Nursing, Department of Medicine, University of Padua, 35131 Padua, Italy; sara.bortot@unipd.it (S.B.); renzo.zanotti@unipd.it (R.Z.)

**Keywords:** functional dependence, assessment, nursing, activities of daily living

## Abstract

Among hospitalised patients, functional decline and increased dependence on others are common health problems. Identifying critical needs is an important starting point to empower patients to improve their own health and behaviour. Once these needs are determined, the most relevant goals for addressing patients’ needs and health potential can be established. This study aimed to test a model for profiling patients using the General Assessment of Hospitalised Patients (ASGO) compared to the Barthel Index (BI) as the gold standard. A retrospective approach was conducted by reviewing administrative data recorded between 2017 and 2020 at the University of Padova, Italy. Data from patients (a) older than 18 years, (b) admitted to a local hospital, and (c) with a stay of at least three days were included in the study. The ASGO and the BI were both used on patients’ admission and discharge from the ward. Data were analysed using STATA software (v.16) (StataCorp. 2019. Stata Statistical Software: Release 16. College Station, TX: StataCorp LLC). The database used for the analysis consisted of 842 patient records, with more than 50% over 75 years of age and consisting mainly of men. The results of the ASGO and the BI were more correlated at discharge (rho = −0.79) than at admission (rho = −0.59). Furthermore, sensitivity and specificity, calculated with reference to the optimal cut-off point (Youden index), demonstrated the highest reliability of the test at discharge (sensitivity: 0.87; specificity: 0.78) compared to admission (sensitivity: 0.77; specificity: 0.72). This result was confirmed by the analysis of the ROC curve: The area under the curve was greater at discharge (89%) than at admission (82%). Analysis of the results obtained from assessments created with the ASGO demonstrates the applicability of this model in the context of hospital care and how well it can represent functional dependence. This study was not registered.

## 1. Introduction

Developments in human resource planning in healthcare facilities have led to studies on the estimation of nursing staff through the creation of patient categorisation systems. Classification systems were created primarily to assess the level of dependence on professional care in hospital wards and to profile patients according to the intensity of nursing care they receive [1]. Much of the current literature agrees that the prospective use of standardised tools for assessing care may represent valid support to improve the organisational appropriateness of healthcare organisations [2].

Assessing the functional status and determining appropriate nursing activities are the first steps in the nursing care process [3]. The functional status of a patient refers to his or her ability to perform activities and tasks that are necessary for daily living and general well-being [4]. It typically considers various aspects such as mobility, self-care abilities, communication skills, cognitive functioning, social interactions, and the ability to perform activities such as dressing, bathing, eating, and managing medications. There are several commonly used tools to measure the functional status of a patient, including, for example, the Activities of Daily Living (ADL) and the Instrumental Activities of Daily Living (IADL) scales [5], the Karnofsky Performance Scale (KPS) [6], the Barthel Index (BI) [7] and the Mini-Mental State Examination (MMSE) [8].

Most models have the characteristic of being specific to the country where they are established, as in the case of diagnosis-related grouping (DRG) [9], especially suitable in an American culture where nurses expect to be involved in decision-making and cost accounting. Since this kind of model has received so much criticism, several efforts have been made to create alternatives that allow needs assessment for nursing care directly on the requirements of patients rather than indirectly on their diagnosis. In the Italian context, for example, the nursing performance information system (SIPI) considers the nurse’s assessment of patient care rather than the amount of time devoted to a specific support activity in order to effectively allocate clinical personnel to nursing tasks [10]. However, this model places more emphasis on the approaching nursing interventions. Other instruments require time to fully understand how to use the tool, which can be quite complex in busy hospital wards [11], as in the case of the RAFAELA tool, designed in Finland during the 1990s. In addition, most models currently in use consider only a small portion of the components of care. In determining the complexity of nursing care, they focus more on the functionality of the subject and the interventions to be administered, while often omitting the subject’s potential for improvement in terms of self-care and competence of the patient or caregiver.

Providing professional care to people with health problems means deliberately including behaviour to activate their self-care potential—if any—or, failing this, to enable new compensatory and adaptive strategies [12]. Assessing a patient’s health potential in relation to their current state of health can be achieved with the General Evaluation of the Hospitalised Patient (ASGO) [13], an easy-to-complete and comprehensive assessment tool used to evaluate patient functioning, which can be managed with supportive care or by empowering the patient. Its application has the potential to provide a weighted profile of the patient to select the use of resources and the type of care plan. The goal is to overcome other conventional valid and reliable methods in which items are equally weighted and are resource intensive, time-consuming, and complex to interpret in a resource-limited clinical setting.

The objective of this study Is to assess the internal and external validity of the ASGO in relation to the BI, which is considered the gold standard for measuring patient functional dependence.

## 2. Materials and Methods

### 2.1. Study Design and Participants

This is a comparative validation study based on a retrospective analysis of consecutive administrative data stored in a database owned and managed by the Laboratory of Studies and Evidence-Based Nursing at Padua University, Italy. Data were recorded in adult patients 18 and over from a local hospital between 1 January 2016 and 1 January 2020. Whilst records of people with severe mental disorders were excluded, from this study it is important to note that over two in five people in the general population have experienced a mental disorder at some time in their life. Data from 842 patients were analysed.

This study consists of two parts: the construction of a hospital patient assessment instrument and the preliminary psychometric evaluation of the instrument. The three-step approach recommended by Clark and Watson was followed [14]. In this regard, conceptualisation (1) consisted of an academic-level debate in which the variables of the instrument were considered according to the definition of the concept of professional nursing. Subsequently, creation of an item pool (2) was obtained in several meetings with the research team. This step involved soliciting and organising the opinions of a panel of professionals with specialised knowledge in nursing science (e.g., clinical nurses, managers, academics) who were intentionally selected to develop and refine the content of the instrument through a series of consensus rounds. The expert group identified and chose each variable’s weighting and ranking through discussion at the same time. The third step involves psychometric evaluation (3), with the goal of validating the ASGO assessment versus the BI. Reliability, validity, and responsiveness (i.e., comparison to a gold standard) were considered for this study in accordance with the COSMIN (Consensus based Standards for the selection of health status Measurement iNstruments) taxonomy [15].

### 2.2. The General Assessment of the Hospitalised Patient (ASGO)

The profile of the patient was based on the ASGO [13], a checklist of 10 non-medical functions weighed by the commitment to support needs. The tool includes mental status, movement/ambulation, circulation, breathing, elimination/sphincter control, feeding, sleep/wake, sensory system, self-care ability, and prevailing mood. The scale was created to retrieve data through direct observation and interviews with a patient and/or other sources of information, such as caregiver(s). Each variable encompasses seven modalities that describe the functional dependence (FD). Table 1 shows the characteristics with the weighting and scoring system. The authors created weights by asking the expert group to rank the variables by priority, where two priorities could be of equal importance (e.g., exercise/walking ability and self-care ability); then, the same group agreed on a ranking system for each variable based on a seven-point scale. The ASGO assessment was available on admission and before hospital discharge. The FD index helps estimate the need for assistance, whether it is substitution, supportive, or basic: the higher the score, the higher the FD of the subject.

### 2.3. The Barthel Index

The BI is a 10-item questionnaire that assesses physical performance in activities of daily living (ADL). It includes feeding, toilet use, bathing, grooming, dressing, bowel and bladder control, chair transfer, climbing, and walking [7]. Each item is scored proportionally, and each level or rank is assigned a certain number of points. The score for each item is determined by the amount of verbal or physical assistance required to complete each task. The score ranges from 0 (total dependence) to 100 (complete independence). The BI had well-established validity and reliability, with the Cohen κ ranging from good (0.61–0.80) to very good (0.81–1.00) and internal consistency (Cronbach α) ranging from good (0.80–0.89) to excellent (0.93) [16]. The Italian culturally adapted BI as a whole has been shown to be valid, reliable, acceptable, easy to understand, and rapidly administrable [17]. For the purpose of this study, BI cut-off scores were defined as ≥60 out of 100 for better functional status and <60 for worse functional status. The BI has been used in the clinical setting in the Veneto region since 2006, as required by law [18]. Evaluation is carried out between 8 and 12 h after admission and at discharge in each patient with at least three days of hospitalisation.

### 2.4. Data Management and Statistical Analysis

The ASGO and the BI were digitalised in REDCap [19], a web-based application for data management and storage. Then, STATA version 16.0 statistical software (StataCorp. 2019. Stata Statistical Software: Release 16. College Station, TX: StataCorp LLC) was used to analyse the data. The gender, age, reason for hospital admission, and length of stay were extracted. Demographic data were presented with descriptive statistics. The ASGO tool was analysed in terms of reliability (Cronbach’s alpha), sensitivity, specificity, and receiver operating characteristic (ROC) curve. Furthermore, the Youden index was adopted to identify the best cut-off point for the instrument. Then statistical analysis to test the correlation between ASGO scores and Barthel Index values was performed using Spearman’s rho coefficient. In order to assess the instrument’s validity, the factor structure of the instrument was assessed by using exploratory factor analysis (EFA) with the principal component analysis (PCA) method. Assumption testing and sampling adequacy were performed first using the Kaiser–Meyer–Olkin (KMO) statistic and Bartlett’s sphericity test.

### 2.5. Ethics

This study was based solely on administrative data, with no patient participation. Thus, consent was not necessary for the secondary use of non-identifiable data that were obtained as part of routine clinical care with the primary goal of quality improvement. According to Italian laws, no formal authorisation was required from an ethics committee. This study was carried out according to the criteria set by the Declaration of Helsinki and the protection of personal data was guaranteed following the Regulation (EU) 2016/679 of the European Parliament.

## 3. Results

Data came from a cohort of 842 hospitalised patients (Table 2). The majority were men (55.0%), with an average 76.4 years of age (SD 12.3). Of these participants, 31.4% were admitted due to a cardiovascular disease, followed by 15.5% with a respiratory disease. The average hospital stay was 20.1 days (SD 13.4). According to the ASGO tool, the average score was 27.1 (SD 10.6) on admission and 24.9 (SD 11.4) at discharge (*p* < 0.001). The mean BI score was lower at admission (mean 39.2, SD 30.3) than at discharge (mean 55.4, SD 33.2). The correlation between paired ASGO scores and BI scores was moderate to strong both for admission (Spearman’s rho = −0.59, *p* < 0.001) and for discharge (Spearman rho =  −0.79, *p* < 0.001) (Table 3).

The Cronbach’s α of the ASGO tool was acceptable both at admission (α = 0.67) and at discharge (α = 0.73) (Table 4).

The optimal cut-off point obtained with the Youden index for the prediction of dependence is an ASGO score of 22.25. At admission, the sensitivity of this cut-off score was 77% and the specificity was 72%. At discharge, the sensitivity for this cut-off score was 87% and the specificity was 78%. All cases with a raw score of <22.25 were classified as “independence”; all other cases were classified as “dependence”.

When the dependence/independence of the ASGO was defined by a cut-off of 22.25, 61% of subjects with total dependence were correctly classified as dependent and 78% of independent subjects were properly classified as independent on admission (Table 5). On the contrary, at discharge, 89% of subjects with total dependence were correctly classified as dependent and 69% of independent subjects were correctly classified as independent.

**Figure 1:**
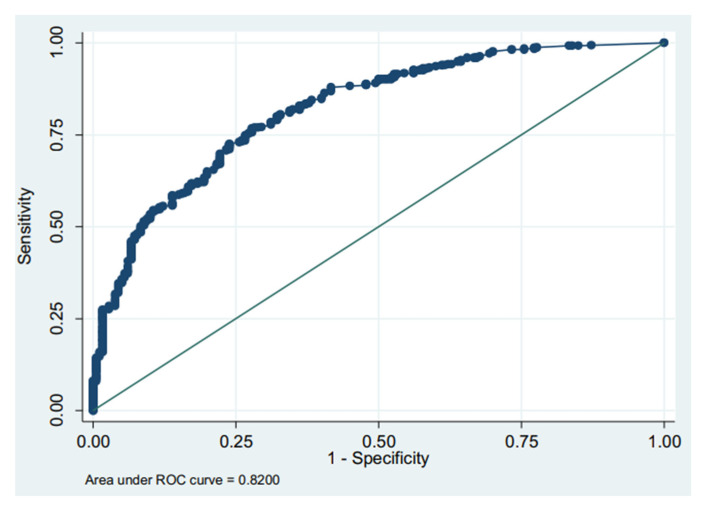
Receiver operating characteristic (ROC) curve of the ASGO on admission.

**Figure 2:**
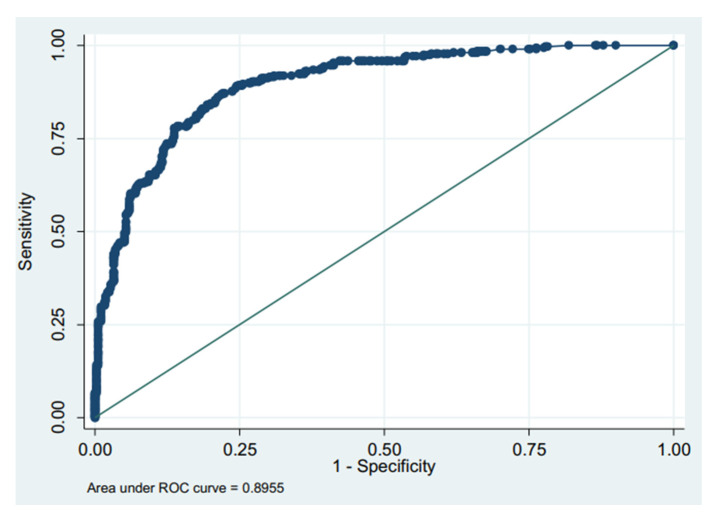
Receiver operating characteristic (ROC) curve of the ASGO at discharge.

The AUC was 0.82 on admission (Figure 1) and 0.89 (Figure 2) at discharge, which demonstrates that the ASGO is an appropriate tool to assess the level of dependence in hospitalised patients compared to the BI. A test with an AUC between 0.80 and 0.90 indicates good discrimination ability [20].

An EFA was performed using a principal component analysis and varimax rotation. Kaiser–Meyer–Olkin sampling adequacy (0.87) confirmed that the data and sample size were adequate for this factor analysis. The Bartlett sphericity test was 2137.8 (*p* < 0.001), which indicates that the correlation matrix was suitable for factoring. The analysis identified two factors that together accounted for 49.51% of the variance (Table 6 and Table 7), whereas the first factor explained 38.64%.

The first factor represents physical functionality, which is composed of bodily functional variables, with self-care and nutrition having the greatest weight. The second factor describes the sensory behaviour and consists mainly of the sensory system and the prevailing mood.

## 4. Discussion

In our study, the FD was derived from the BI, considered the gold standard of evaluation. The ASGO instrument showed good levels of concurrent validity when correlated with the BI score obtained. The ASGO and BI scores were more correlated at discharge (rho = −0.79) than on admission (rho = −0.59). Furthermore, the ASGO scale revealed more sensitivity (the ability of the tool to detect the dependence of a true patient) at discharge compared to on admission (0.89 vs. 0.61), whereas more specificity (the ability of the tool to detect the independence of a true patient) was detected on admission than at discharge (0.78 vs. 0.69). These results were confirmed by analysis with the ROC curve.

There are several methods to profile patient characteristics—for example, those that incorporate medication regimen complexity, cognition, physical and mental health, hospitalisation, and physical function [10,11]. Others use algorithms that classify patients into homogeneous groups according to physical function and costs calculated by reimbursement systems [1]. None of the reported methods are per se wrong. However, what is generally lacking is a model that can also include the potential of the patient in relation to their current state of health.

Our results suggest that the use of both the BI and the ASGO would allow for a similarly effective evaluation. However, the ASGO tool may offer some advantages. First, the BI provides a limited scoring system. The 10 items have the following scoring combinations: (a) 0 and 5; (b) 0, 5, and 10; or (c) 0, 5, 10, and 15. In comparison, the ASGO score uses a seven-point scale for each variable, allowing for subtle improvements or declines in certain activities. When looking at the variable of feeding as an example, the ranking is as follows: free (0.6), enteral nutrition (1.2), parenteral nutrition (1.8), mouth ulcers/infections (2.4), difficult/painful swallowing (3.0), limited/absent chewing (3.6), and spoon-fed/direct eating help (4.2). Although it is not universally clear how to weight the individual elements of a scale, tools that do not consider how the elements can be weighted differently can result in a biased estimate of the measured object [21].

Furthermore, the BI does not account for cognitive impairments or mental states that may affect a person’s ability to perform daily tasks effectively. On the contrary, mental status is the first variable assessed by the ASGO tool, which acquired the highest weighted score (1.6 of 10) in the general evaluation of the hospitalised patient. The relationship between the mental state of a patient, the activities of daily living performance, and the nurses’ workload is a well-recognised topic in the literature. Impaired mental health can disrupt executive functioning, which involves higher-level cognitive processes such as planning, organising, and initiating tasks [22]. Furthermore, mental status, which may include symptoms of agitation, aggression, and confusion, significantly influences workload by increasing the need for observation, intervention, and participation [23].

Lastly, the BI differs from the ASGO in that it does not consider self-care abilities. In fact, “self-care ability” had the second highest weighted score on the test (1.3 of 10). The self-care ability of a patient is of great importance when it comes to activities of daily living. This ability is crucial for personal autonomy, rehabilitation, prevention of complications, social participation, and supporting the well-being of both patients and their caregivers. Furthermore, the relationship between self-care capacity and the demand for nursing care is inversely related [24].

The analysis of the structure in our study revealed two factors that can be associated with the two main characteristics of patient status: physical functionality and sensory behaviour. This is despite the fact that the percentage of variance explained cannot be considered satisfactory. From a theoretical point of view, the ASGO instrument provides a weighted profile that provides a quantitative and qualitative description of a hospitalised patient [12]. Its application helps nurses make decisions about the use of resources and the type of patient care. The ASGO reflects a single basic concept that maps all of the patient’s physical and cognitive functions, exploring potential subject variability with a tiered checklist (i.e., seven modalities for each variable).

### Strengths and Limitations

The conclusions of this study are preliminary and should be validated through additional research and multi-institutional investigations. In fact, the ASGO tool lacks comprehensive validation in terms of its psychometric properties and requires further studies to establish its robustness and reliability. The study’s key shortcoming is that the gold standard for defining the true level of dependence may not be perfect (e.g., lack of cultural and contextual specificity). However, this reference was the best available, as prescribed by the Italian legislator, to evaluate the degree of dependence of the patient and, consequently, the complexity of the care activities to be dedicated to a patient. It might be helpful to use other comparative instruments in the future (e.g., the Instrumental Activities of Daily Living Scale). In addition, the study involved patients admitted to various departments, not considering, for example, a subgroup analysis. There are some settings (e.g., surgical wards) where functional autonomy varies rapidly before and during hospitalisation and at the time of discharge. Lastly, since the evaluation is typically conducted by an observer (both for the BI and for the ASGO), there is a potential bias in rating an individual performance, which could affect the reliability and precision of the results.

The study also has some notable strengths. The study participants formed a large sample with all representative medical problems. In addition, this comparative study found good agreement in measuring the degree of patient dependence between the two instruments. These results provide the starting point for a subsequent full evaluation of the ASGO instrument.

## 5. Conclusions

Our findings suggest that it is feasible to measure the FD of a hospitalised patient by using the ASGO tool. Moreover, the two instruments achieved similar effective assessments. However, compared to the BI, the ASGO instrument also considered patient mental status and self-care ability. To our knowledge, there are few studies in the nursing field that attempt to rank patients using a weighted ranking system that allows subtle improvements or deteriorations in specific activities to be captured. Future research is essential to validate the tool and establish its clinical utility. This tool could be used as a support tool in organisational management in terms of the rapid identification of patients who need more nursing assistance and allocation of resources.

## Figures and Tables

**Table 1:** Weights and scores of the General Assessment of Hospitalised Patient tool.

Variable	Variable Weight	Ranking
Mental status	1.6	Awake and lucid (1.6)—non-responsive (3.2)—comatose (4.8)—disoriented behaviour (6.4)—slowed thinking (8.0)—memory loss (9.6)—forgetfulness and confusion (11.2)
2. Movement/ambulation	1.3	Full movement (1.3)—walking aids (2.6)—autonomous wheelchair user (3.9)—bedridden (5.2)—bedridden with risk of falling (6.5)—supported by someone (9.1)
3. Circulation	0.4	Adequate (0.4)—oedemas (0.8)—cold (1.2)—hypotension (1.6)—fatigue (2.0)—erythema (2.4)—skin ulcers (2.8)
4. Breathing	0.4	Adequate (0.4)—tracheostomy (0.8)—mechanical ventilation (1.2)—body position required (1.6)—self-management of respiratory therapy (2.0)—fatigue (2.4)—impaired, with O_2_ support (2.8)
5. Elimination/sphincters control	1.2	Normal (1.2)—urinary catheter (2.4)—faecal incontinence (3.6)—urinary incontinence (4.8)—partial urinary control (6.0)—faecal partial control (7.2)—enterostomy (8.4)
6. Feeding	0.6	Free (0.6)—enteral nutrition (1.2)—parenteral nutrition (1.8)—mouth ulcers/infections (2.4)—difficult/painful swallowing (3.0)—limited/absent chewing (3.6)—spoon-fed/direct eating help (4.2)
7. Sleep/wake	1.2	Regular (1.2)—sleep medicine (2.4)—regular bedtime routine (3.6)—tiredness (4.8)—frequent awakenings (6.0)—unrefreshing sleep (7.2)—disturbed sleep, apnoea (8.4)
8. Sensory system	0.6	Normal (0.6)—hearing impaired (1.2)—visually impaired (1.8)—deaf (2.4)—blind (3.0)—environmental perception impaired (3.6)—pain (4.2)
9. Self-care ability	1.3	Complete (1.3)—minimal bathing assistance (2.6)—major bathing assistance (3.9)—unable to dress (5.2)—passive movement (6.5)—following movements (7.8)—basic moves (9.1)
10. Prevailing mood	1.4	Stable (1.4)—needs to be encouraged (2.8)—social withdrawal (4.2) emotional stress (5.6)—anxiety (7.0)—apathy (8.4)—depression, suicidal ideation (9.8)

Note: scoring system: functional dependence (FD) = Σ(Ranking × Variable weight)/10. Patient’s profiles: minimum care required (1.1–2.59), medium care required (2.6–3.69), high care required (3.7–4.79), and maximum care required (≥4.8). The variables of circulation, feeding, elimination/sphincter control, and sensory system allow more than one rank to be indicated.

**Table 2:** Sample characteristics (n = 842).

Variable	n = 842
Gender, n (%)	
Female	379 (45.0)
Male	463 (55.0)
Age (years), mean (SD)	76.4 (12.3)
Reason for admission to the hospital, n (%)	
Cardiovascular disease	264 (31.4)
Respiratory disease	131 (15.5)
Trauma	103 (12.2)
Gastrointestinal disease	89 (10.6)
Cerebrovascular disease	66 (7.8)
Cancer	62 (7.3)
Endocrine disorders	45 (5.3)
Miscellaneous	82 (9.9)
Length of stay in hospital (days), mean (SD)	20.1 (13.4)

SD, standard deviation.

**Table 3:** Spearman correlation coefficients between ASGO scores and the Barthel Index.

Timing	ASGO Mean (SD)	Barthel Index Mean (SD)	Spearman’s Rho	*p*-Value
On admission	27.1 (10.6)	39.2 (30.3)	−0.5916	<0.001
Before discharge	24.9 (11.4)	55.4 (33.2)	−0.7988	<0.001

ASGO, General Assessment of Hospitalised Patient; SD, standard deviation. Note: Negative values are interpreted as a positive correlation since the scales are graduated inversely.

**Table 4:** The reliability of the ASGO.

Timing	Range of Score	Mean	SD	*p*-Value	Cronbach’s α
On admission	10.0–66.1	27.1	10.6	<0.001	0.67
At discharge	10.0–61.2	24.9	11.4		0.73

ASGO, General Assessment of Hospitalised Patient; SD, standard deviation.

**Table 5:** Patient dependence assessment results, paired.

On Admission	Barthel Index < 60 Dependence	Barthel Index ≥ 60 Independence	Total
ASGO ≥ 22.5 dependence	130	126	256
ASGO < 22.5 independence	82	454	536
Total	212	580	792
**At Discharge**	**Barthel Index < 60** **Dependence**	**Barthel Index ≥ 60** **Independence**	**Total**
ASGO ≥ 22.5 dependence	333	129	462
ASGO < 22.5 independence	41	289	330
Total	374	418	792

ASGO, General Assessment of Hospitalised Patient. Note: On admission, sensitivity = n of positive tests/n of dependent subjects = 130/212 = 0.61; specificity = n of negative tests/n of independent subjects = 454/580 = 0.78. At discharge, sensitivity = n of positive tests/n of dependent subjects = 333/374 = 0.89; specificity = n of negative tests/n of independent subjects = 289/418 = 0.69.

**Table 6:** Results of exploratory factor analysis (extraction method: principal component analysis) (n = 842).

Component	Initial Eigenvalues			Extraction Sums of Squared Loadings		
	Total	% of Variance	Cumulative %	Total	% of Variance	Cumulative %
1	3.86	38.64	38.64	3.86	38.64	38.64
2	1.08	10.87	49.51	1.08	10.87	49.51
3	0.96	9.60	59.11			
4	0.76	7.65	66.77			
5	0.72	7.24	74.02			
6	0.66	6.66	80.68			
7	0.56	5.63	86.32			
8	0.52	5.24	91.56			
9	0.48	4.87	96.44			
10	0.35	3.56	100.00			

**Table 7:** Principal component analysis using Varimax rotation.

Items	Factor 1	Factor 2
Mental status	**0.640**	0.277
Movement/ambulation	**0.683**	0.231
Circulation	0.250	**0.557**
Breathing	0.250	**0.604**
Elimination/sphincter control	**0.696**	0.059
Feeding	**0.707**	0.164
Sleep/wake	**0.434**	0.142
Sensory system	0.090	**0.786**
Self-care ability	**0.724**	0.414
Prevailing mood	0.196	**0.749**

## Data Availability

The de-identified data underlying the results presented in this study are available upon request to the corresponding author.

## Public Involvement Statement

There was no public involvement in any aspect of this research.

## Guidelines and Standards Statement

Data are reported here according to the STrengthening the Reporting of OBservational studies in Epidemiology (STROBE) statements to improve truthfulness and ensure clarity. “https://www.equator-network.org/reporting-guidelines/strobe/” (accessed on 10 June 2023).
